# Mitochondrial 3 beta-hydroxysteroid dehydrogenase (HSD) is essential for the synthesis of progesterone by corpora lutea: An hypothesis

**DOI:** 10.1186/1477-7827-3-11

**Published:** 2005-04-03

**Authors:** John C Chapman, Jose R Polanco, Soohong Min, Sandra D Michael

**Affiliations:** 1 Department of Biological Sciences, Binghamton University, Binghamton, NY 13902-6000, USA; 2 Notre Dame Ambulatory Care Center, Medical Director, 1000 Broad Street, Central Falls, RI 02863, USA

## Abstract

In mouse ovaries, the enzyme 3 beta-hydroxysteroid dehydrogenase (HSD) is distributed between microsomes and mitochondria. Throughout the follicular phase of the estrous cycle, the HSD activity in microsomes is predominant; whereas, after LH stimulation, HSD activity during the luteal phase is highest in the mitochondria. The current study examined whether or not LH stimulation always results in an increase in mitochondrial HSD activity. This was accomplished by measuring the HSD activity in microsomal and mitochondrial fractions from ovaries of pregnant mice. These animals have two peaks of LH during gestation, and one peak of LH after parturition. It was found that mitochondrial HSD activity was highest after each peak of LH. It is proposed that mitochondrial HSD is essential for the synthesis of high levels of progesterone. The increase in HSD activity in mitochondria after LH stimulation occurs because: 1) LH initiates the simultaneous synthesis of HSD and the cholesterol side-chain cleavage enzyme (P450scc); and, 2) HSD and P450scc bind together to form a complex, which becomes inserted into the inner membrane of the mitochondria. High levels of progesterone are synthesized by mitochondrial HSD because: 1) the requisite NAD+ cofactor for progesterone synthesis is provided directly by the mitochondria, rather than indirectly via the rate limiting malate-aspartate shuttle; and, 2) the end-product inhibition of P450scc by pregnenolone is eliminated because pregnenolone is converted to progesterone.

## Background

With the exception of 3β-hydroxysteroid dehydrogenase (HSD), the enzymes involved in the conversion of cholesterol to steroid hormones are located in either the mitochondria or the endoplasmic reticulum. HSD is unique in that it is located in both subcellular organelles. In either location, HSD converts pregnenolone and dehydroepiandosterone (DHEA) to progesterone and androstenedione, respectively, using NAD^+ ^as cofactor. The reason for two separate sites for this enzyme is not known.

Establishing the existence of two separate locations for HSD has been a lengthy process. In 1956, Beyer and Samuels reported that the microsomal (endoplasmic reticulum) and mitochondrial fractions from the homogenate of bovine adrenal cortex contained HSD activity [1]. However, the HSD activity found in the mitochondrial fraction was attributed to microsomal contamination and the result of the homogenizion process. While this study established the legitimacy of microsomal HSD, it tended to preclude further research on mitochondrial HSD. For mitochondrial HSD to be considered a distinct and separate entity, additional research over a number of years would be required. Starting in 1965, investigators began to report a dual location for HSD in ovaries [2-4], testes [5,6], human term placenta [7-10], and rat adrenal cortex [11-14]. In toto, these studies suggested that mitochondrial HSD was indeed a separate entity. Other investigators, however, still considered mitochondrial HSD activity to be due to microsomal contamination [15,16], and the result of a redistribution artifact [17].

In 1979, we reported the results of an intracellular enzyme distribution study of HSD, cytochrome c oxidase (mitochondrial marker), and steroid 21 hydroxylase (microsomal marker) in rat adrenal cortex [18]. We found that exhaustively washed mitochondria retained 26 % of total HSD activity. In retrospect, this percentage appears to be on the low side. For example, when the specific activity of microsomal HSD is determined, and its contribution to the HSD activity in the remaining cell fractions (nuclear/unbroken cell, mitochondrial, and mitochondrial wash fractions) ascertained, then a maximum of 60 % of total homogenate HSD activity can be attributed to microsomal HSD. This indicates that mitochondrial HSD constitutes 40 % of total homogenate HSD activity, rather than 26 % as we initially reported. In rhesus monkey placenta, HSD activity is equally distributed between mitochondria and microsomes [19], and in bovine adrenal cortex, mitochondrial HSD comprises 30 % of total HSD activity [20,21].

Our study also found that mitochondrial HSD utilizes matrix space NAD^+ ^as cofactor, indicating that the enzyme is located in the inner mitochondrial membrane [18]. This location for mitochondrial HSD has been established in bovine adrenal cortex [20,21], and in rat testis [5]. The combined techniques of immuno-cytochemistry and electron microscopy have identified immune reactive HSD in mitochondria of human ovary [22], and in the mitochondria of rat ovary, testis, and adrenal cortex [23]. Mitochondrial HSD has now been isolated from bovine adrenal cortex [21,24], and from human term placenta [25,26], and purified to homogeneity. It is now known that microsomal HSD and mitochondrial HSD are identical proteins [25-28]. The reason for two locations for the same enzyme has yet to be determined.

In a study of the intracellular distribution of mitochondrial HSD and microsomal HSD in mouse ovaries over the course of the estrous cycle [29], we reported that during diestrus (luteal phase), the specific activity of mitochondrial HSD was 80 % higher than that of microsomal HSD. This is in sharp contrast to the other three stages of estrus where microsomal HSD had the highest specific activity. In a study in which the cDNA of human placental HSD was transfected into Sf9 cells, the resultant HSD enzyme was distributed between mitochondria and microsomes [27]. In the luteal cells of mouse ovary, the distribution of the enzyme is skewed in favor of the mitochondria. During the luteinization process, luteal cells express high levels of mRNA for the cholesterol side-chain cleavage enzyme (P450_scc_) and for HSD [30,31]. The simultaneous synthesis of these two enzymes is very likely the reason for the increase in mitochondrial HSD activity, as will be discussed later.

The results of our previous study [29] tentatively suggested that the increase in mitochondrial HSD activity in mouse ovary during diestrus is due to LH. The pregnant mouse has two peaks of LH during gestation, and one peak of LH after parturition [32]. After each peak of LH, the levels of circulating progesterone increase [33]. The present study examined the distribution of HSD activity in pregnant mouse ovaries to determine whether or not mitochondrial HSD activity also increased after each peak of LH. We found that it did, which led to our proposed explanation for the reason for two separate locations for HSD in corpora lutea.

## Methods

### Animals

Female mice of the (C3H/HeJ × 129/J)F_1 _(C31) hybrid were used in the study. Parental stocks were purchased from Jackson Laboratories, Bar Harbor, ME. The mice were housed in an animal room kept at 24°C, with controlled lighting of 14L:10D (lights on at 0600 hr and off at 2000 hr). Purina lab chow (Ralston-Purina, St. Louis, MO) and water were provided ad libitum. The C31 offspring were weaned at 23–28 days of age. Female siblings were housed two per cage. For pregnancy experiments, C31 females were mated with C31 males. Only those with regular estrous cycles (4–5 days) were used for the study.

### Determining Stage of the Estrous Cycle and mating

In order to promote a regular estrous cycle in the C31 females, cage shavings were taken from cages containing C31 males, and placed in cages containing the females. Also, male cages surrounded female cages. Vaginal smears were taken daily, usually in the afternoon. The smears were spread on a glass slide in a drop of physiological saline, stained using hematoxylin and eosin Y, and the stage of estrous determined by the method of Rugh [34].

In all experiments, each study group consisted of six females that were between 80 and 190 days of age. Two of the study groups were comprised of animals that were in diestrus and proestrus. The remaining groups all consisted of pregnant animals. These groups were formed as follows: females that were in either proestrus or estrus were mated late in the afternoon or about 2 hr prior to lights off. At lights on the following morning, and for subsequent days if necessary, all females were inspected for the presence of vaginal plugs. The day of vaginal plug was considered as day 0 of pregnancy. Over the gestation period, pregnant females were sacrificed on days 5, 10, 15, and 20. An additional group of females was sacrificed on day 5 postpartum.

### Tissue Collection and Processing

Animals were lightly etherized, weighed, and then decapitated. The ovaries were removed, trimmed of fat, weighed in pairs, and placed on an ice-filled petri dish in a few drops of homogenizing buffer (0.3 % BSA, 1 mM EDTA, 0.25 M Sucrose, and 30 mM Tris-HCL [pH 7.4], 4°C), as per Chapman and Sauer [18]. The twelve ovaries were finely minced, then transferred to an ice-cold 5 ml Potter-Elvehjem glass homogenizer. The volume of the sample was brought up to 4 ml with additional homogenization medium, and the ovaries homogenized in the cold room (4°C) with 8 complete strokes of a motorized Teflon pestle. The homogenate was then transferred to a 10 ml centrifuge tube, and the glass homogenizer rinsed with 1 ml of homogenizing medium. The rinse was combined with the homogenate, and a 1 ml sample of the total homogenate removed and saved for later assay.

The uteri were removed from 10 day, 15 day, and 20 day pregnant mice. Trophoblasts were excised from the middle section of each uterine horn. After weighing, the trophoblasts were minced, and processed as described for the ovaries, except that the mince was homogenized in a Thomas glass-glass homogenizer.

### Differential Centrifugation

The ovarian homogenate, contained in a 10 ml centrifuge tube, was centrifuged at 700 × g in a Sorvall RC-5 refrigerated centrifuge for 10 min. The supernatant was removed and spun at 10,000 × g for 20 minutes in the same centrifuge. The low-speed pellet, containing nuclei and unbroken cells, was resuspended in 1.5 ml homogenizing buffer. Following the 10,000 × g centrifugation, the resultant mitochondrial pellet was resuspended in 1.5 ml homogenizing buffer. The postmitochondrial supernatant was centrifuged in a Beckman L65 Ultracentrifuge (Beckman Co., Fullerton, CA) for 1 h at 105,000 × g. This yielded cytosol and a pellet of microsomes. Microsomes were resuspended in 1.5 ml homogenizing buffer using a 2 ml Potter-Elvehjem glass homogenizer with Teflon pestle. All fractions, including the total homogenate, were divided into aliquots in 12 × 75-mm borosilicate glass tubes, covered with Parafilm (American Can Co., Greenwich, CT), and frozen at -10°C. Enzymatic analyses were scheduled so that the frozen sub-cellular fractions were thawed only once prior to assay.

### Enzymatic Analyses and Other Assays

The total homogenate, low speed pellet (nuclei/unbroken cells), mitochondrial, and microsomal fractions were assayed for HSD activity by measuring the conversion of pregnenolone to progesterone. Duplicate tissue samples of 50 μl and 100 μl were added to 15-ml glass test tubes containing 1 ml of incubation medium (50 mM sucrose, 20 mM KC1, 1 mM EDTA, 30 mM Tris-HCl [pH 7.4], 0.3% BSA), and 0.5 mM NAD^+^, as per Chapman et al. [29]. The incubates were then placed in a 37°C water bath and allowed to equilibrate for 5 min. The reaction was started by the addition of 100 nmol of pregnenolone in 10 μl of ethanol. After 15 min of incubation, the progesterone product was extracted into 1 ml of spectral grade heptane. The absorbency of progesterone was measured in a Gilford Response spectrophotometer (Gilford Systems, Oberlin, OH). Progesterone has an absorbency peak at 233 nm in heptane and a molar extinction coefficient of 17,000 [18]. In order to access the extraction efficiency of 1 ml of heptane, known concentrations of progesterone standards were run concurrently with the tissue samples. After extraction into heptane, the absorbancy of the standards was compared to their absorbance measured directly in heptane.

Cytochrome c oxidase, an inner mitochondrial membrane marker, was assayed by the procedure of Wharton and Tzalgaloff [35]. Enzymatic activity was determined by the rate of decrease in absorbancy at 550 nm. Protein content was measured by using the method of Bradford [36]. All assays were in duplicate. Replicate data were analyzed for significant differences using ANOVA (Dunnett, and Scheffe' F-test).

## Results

**Figure 1:**
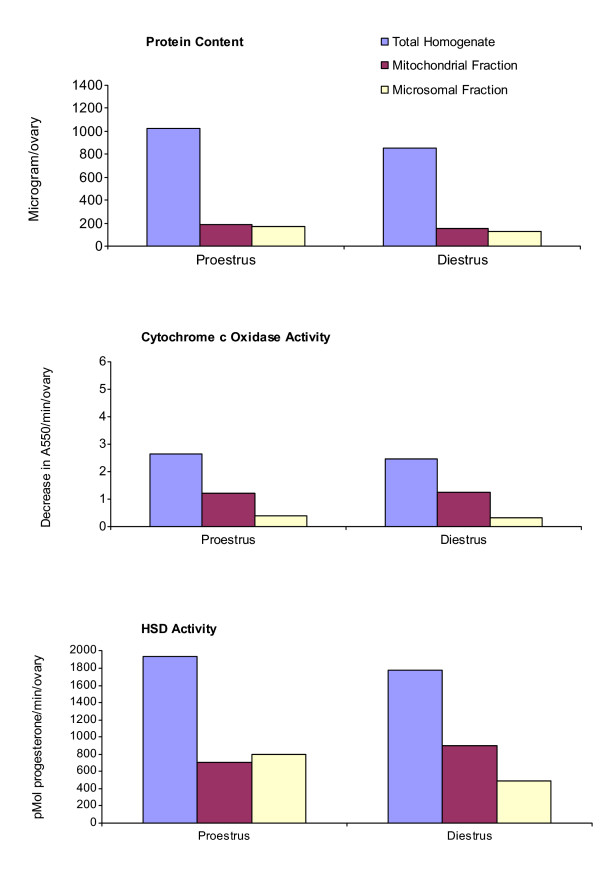
**Protein content, cytochrome c oxidase activity, and HSD activity in total homogenates, and in mitochondrial and microsomal fractions from ovaries of C31 mice that were in either proestrus or diestrus**. Each experimental group consisted of 6 mice. Mitochondrial and microsomal fractions were isolated by differential centrifugation. Results are expressed per individual ovary.

The distribution of HSD activity between mitochondria and microsomes undergoes a unique shift in the transition from proestrus to diestrus. At proestrus, for example, the highest HSD activity is in the microsomes. At diestrus, in contrast, mitochondrial HSD activity is almost double that of microsomal HSD [29]. The present study re-examined this phenomenon; and, as Figure 1 shows, the activity of mitochondria HSD increases significantly at diestrus. The activity of the mitochondrial inner membrane enzyme, cytochrome c oxidase, also increases at diestrus. Total ovarian protein, in contrast, decreases.

**Figure 2:**
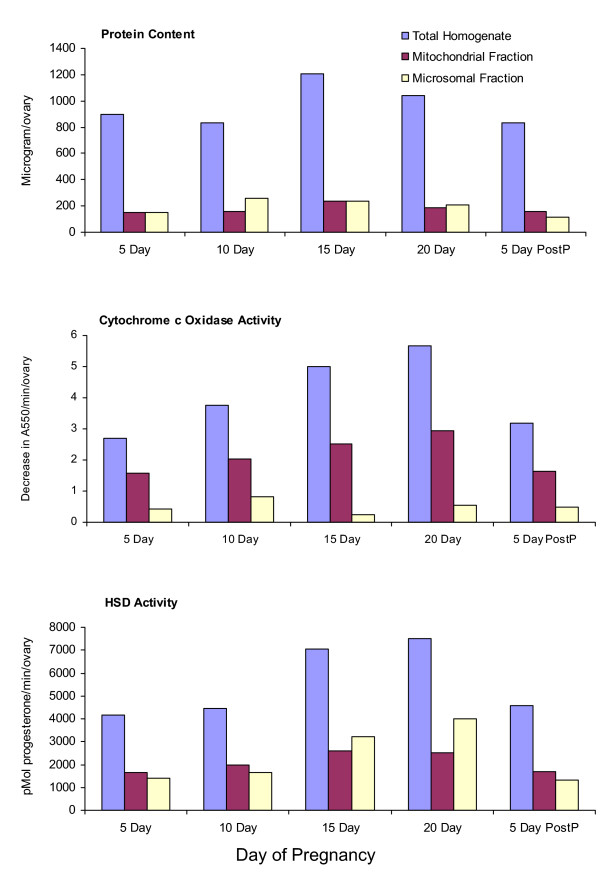
**Protein content, cytochrome c oxidase activity, and HSD activity in total homogenates, and in mitochondrial and microsomal fractions from ovaries of C31 mice that were pregnant for 5 days, 10 days, 15 days, and 20 days, or were 5 days postpartum**. Each experimental group consisted of 6 mice. Mitochondrial and microsomal fractions were isolated by differential centrifugation. Results of two separate experiments are expressed per individual ovary. Statistical analyses of the enzymatic activities of cytochrome c oxidase and HSD in total homogenates at each time-point showed a significant difference @ P < .05, when compared to animals in diestrus. In addition, the HSD activities in all mitochondrial and microsomal fractions of pregnant mice were significantly different @ P < .05, when compared to animals in diestrus.

Figure 2 contains the results of two separate experiments in which HSD activity was measured in mitochondrial and microsomal fractions during pregnancy, and 5 days after parturition. As indicated, the activities of mitochondrial HSD and microsomal HSD both increased over the course of the gestation period. However, the increase in HSD activity in the two organelles was inconsistent. For example, at 15 days and 20 days of gestation, the highest HSD activity was in the microsomal fraction. In contrast, at 5 days and 10 days of gestation, and at 5 days postpartum, mitochondrial HSD activity was greater than that of microsomal HSD. These three time points directly follow the peaks of LH [32]. Cytochrome c oxidase activity also increased during pregnancy. At day 20, cytochrome c oxidase activity was more than double the activity measured at 5 days. Total ovarian protein was inversely correlated with the peaks of LH. For example, at 15 days and 20 days of gestation, each ovary contained 1190 μg protein and 1025 μg protein, respectively. In contrast, at 5 days and 10 days of gestation, and at 5 days postpartum, each ovary contained 890 μg protein, 825 μg protein, and 810 μg protein, respectively. This relationship between LH and total ovarian protein also occurs during the estrous cycle [29]. For example, as shown in Figure 1, each ovary at proestrus contained 1030 μg protein; whereas at diestrus, each ovary contained 838 μg protein.

**Figure 3:**
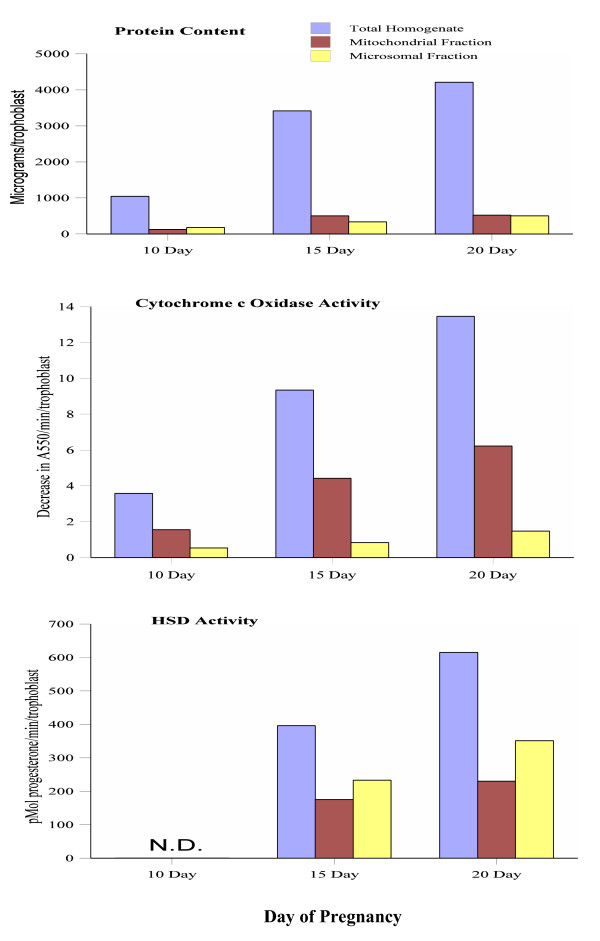
**Protein content, cytochrome c oxidase activity, and HSD activity in total homogenates, and in mitochondrial and microsomal fractions from trophoblasts of C31 mice that were pregnant for 10 days, 15 days, and 20 days**. Each experimental group in the 10 day and 20 day pregnant animals consisted of 6 mice. N = 12 for the 15 day pregnant group. Mitochondrial and microsomal fractions were isolated by differential centrifugation. Results are expressed per individual trophoblast. N.D. = Not detected.

Figure 3 contains the results of the measurement of HSD activity in mitochondrial and microsomal fractions of trophoblasts from 10 day, 15 day, and 20 day pregnant mice. As indicated, HSD activity was not detected (N.D.) in trophoblasts from 10 day pregnant mice. However, trophoblasts from 15 day and 20 day pregnant mice were found to produce 0.4 nmol progesterone/min/trophoblast and 0.6 nmol progesterone/min/trophoblast, respectively. Note that the highest HSD activity in trophoblasts is in the microsomal fraction.

## Discussion

The results of this and the previous study [29] leave little doubt that mitochondrial HSD activity increases after LH stimulation. How the increase in mitochondrial HSD activity is achieved and for what purpose, are the topics of this discussion.

When mitochondrial HSD was initially purified from the bovine adrenal cortex, the enzyme was found to have a close association with P450_scc _[24]. This association was of such a high degree that mitochondrial HSD actually copurified with P450_scc_. Antibodies against mitochondrial HSD precipitated both HSD and P450_scc_; and, conversely, antibodies against P450_scc _precipitated both P450_scc _and mitochondrial HSD. The degree of association between the two enzymes was measured, and a binding constant (K_D_) of 0.12 μM was determined. As would be expected, P450_scc _also bound to purified microsomal HSD [24]. HSD is insoluble and inactive in an aqueous medium, due to a segment of the protein, referred to as the "membrane-spanning domain" [27]. Delete this segment and HSD becomes soluble. With the segment in place, HSD is inserted into the membranes of microsomes and mitochondria [27]. The observation that mitochondrial HSD activity increases after LH stimulation suggests that HSD is preferentially inserted into the mitochondrial membrane. The fact that HSD binds to P450_scc _is very likely the mechanism for its insertion. This suggests that HSD and P450_scc _bind together, either during, or directly after their synthesis, since it is unlikely that HSD could bind to P450_scc_, already in place. The mRNAs for HSD and P450_scc _are expressed concurrently in luteal cells of the rat [37-40], cow [41,42], sheep [42-44], horse [45], macaque monkey [46], and human [38,47-49].

**Figure 4:**
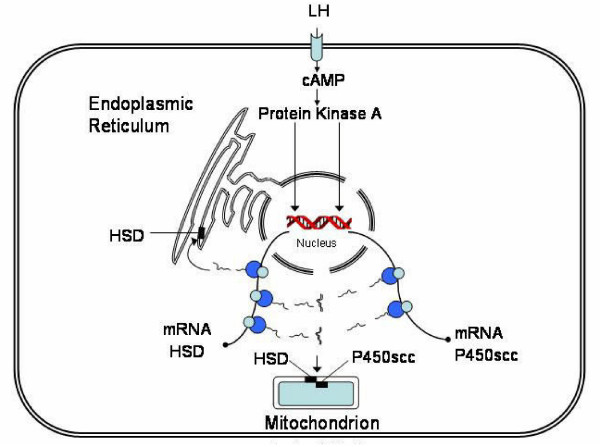
**Representative diagram of the proposed effect of the concurrent synthesis of HSD and cytochrome P450_scc _on the intracellular distribution of HSD in luteal cells**. Initiated by LH; the transcription of HSD mRNA and P450_scc _mRNA results in the simultaneous production of HSD and P450_scc_. The two enzymes bind to each other to form a complex, which is then inserted into the inner mitochondrial membrane. Molecules of HSD that do not bind to P450_scc _are inserted into the membrane of the endoplasmic reticulum.

Figure 4 is a representative diagram of the proposed effect of the concurrent synthesis of HSD and P450_scc _on the intracellular distribution of HSD in luteal cells. Initiated by LH; the expression of HSD mRNA and P450_scc _mRNA results in the simultaneous synthesis of HSD and P450_scc_. The two enzymes bind to each other to form a complex, which is then inserted into the inner mitochondrial membrane. Molecules of HSD that do not bind to P450_scc _are inserted into the membrane of the endoplasmic reticulum.

In the mouse there are peaks of estradiol-17β at proestrus (100 pg/ml) and metestrus (200 pg/ml) [29]. However, these levels are significantly less than the levels of progesterone produced throughout the luteal phase. During pregnancy the levels of circulating progesterone are even higher. One could argue that the increased levels of circulating progesterone in pregnant mice are due to the HSD activity in trophoblasts. This possibility was addressed in the current study. As shown in Figure 3, homogenates of individual trophoblasts from 15 day and 20 day pregnant mice are capable of producing 0.4 nmol progesterone/min/trophoblast and 0.6 nmol progesterone/min/trophoblast, respectively. This level of HSD activity in a single trophoblast is only 3 % of the HSD activity produced by the paired ovaries. However, large litters would increase the percentage. At day 10 there is no question that the ovaries are the major source of circulating progesterone, which averages 55 ng/ml [33]. This level of progesterone is between 275 fold and 550 fold of the peak levels of estradiol-17β produced during the follicular phase.

In order for luteal cells to synthesize high levels of progesterone, a number of events have to occur. First, higher levels of the two enzymes, P-450_scc _and HSD, have to be produced. This event is initiated when their respective mRNAs are expressed, as referenced above. Secondly, the increase in steroid synthesis requires an increased supply of cholesterol. This is achieved by removing cholesterol from cholesterol ester stores [50,51], and by initiating the de novo synthesis of cholesterol [52-54]. The latter event is quite likely in anticipation of fertilization of the ova and the need for a supply of cholesterol beyond diestrus. In the pregnant mouse, luteal cells synthesize high levels of progesterone for three weeks [33]. The de novo synthesis of cholesterol requires a carbon source, as well as ATP and NADPH. In luteal cells the carbon source is acetate; ATP is generated through glycolysis; and NADPH is produced during the oxidative decarboxylation of isocitrate and malate [54]. The enzymes that catalyze the latter reaction are NADP^+^-linked isocitrate dehydrogenase and NADP^+^-linked malate dehydrogenase. Both enzymes are in abundance in the cytoplasm of luteal cells [55].

The enzyme, P450_scc _is capable of producing 53 nmol pregnenolone/min/mg protein [56]. This requires an undiminished supply of NADPH, as well as the aforementioned cholesterol. The NADPH that is produced in the cytoplasm is of no direct use to the P450_scc _enzyme. However, reducing equivalents from NADPH can be transferred from the cytoplasm to mitochondria via the malate-aspartate shuttle [57,58]. Unfortunately, the mitochondria in luteal tissue lack an NADP^+^-linked malate dehydrogenase [59,60]. As a result, NADPH cannot be generated in the mitochondria by the oxidative decarboxylation of malate. This is in sharp contrast to adrenal cortex tissue, which does have a mitochondrial NADP^+^-linked malate dehydrogenase [61,62]. In luteal tissue the mitochondria and cytoplasm both contain an NAD^+ ^-linked malate dehydrogenase, each enzyme having a high specific activity [55,59]. This indicates that there is ample capacity to transfer reducing equivalents from NADH into the mitochondria. If this transfer were to occur in tissues such as liver and heart, the NADH would be used to produce ATP. In steroidogenic tissues, the NADH can also be used to produce NADPH. For example, in mitochondria of luteal tissue, reducing equivalents are transferred from NADH to NADP^+ ^by an energy-independent pyridine-nucleotide transhydrogenase [56]. Sonicates of luteal mitochondria are reported to catalyze the production of 60 nmol NADPH/min/mg protein from NADH [56]. Mitochondria in adrenal cortex tissue have a similar process, except that the transfer is energy-dependent [63]. The main source of NADPH in the mitochondria of luteal tissue is provided by NADP^+^-linked isocitrate dehydrogenase [56]. This enzyme is capable of reducing 253 nmol NADP^+^/min/mg protein [56].

In the synthesis of progesterone, NAD^+ ^is reduced to NADH. In order to maintain a high rate of progesterone synthesis, NADH has to be continually oxidized to NAD^+^. With microsomal HSD, the oxidation of NADH would have to occur via either the α-glycerol phosphate shuttle or the malate-aspartate shuttle. Both shuttles transfer reducing equivalents from NADH into the mitochondria. However, the α-glycerol phosphate shuttle does not operate in the ovary [64], which leaves the transfer of reducing equivalents to the malate-aspartate shuttle. The ovary already heavily utilizes this shuttle. As discussed earlier, the malate-aspartate shuttle transfers reducing equivalents from NADH into the mitochondria for the P450_scc _enzyme. In addition, the shuttle is involved in the oxidation of the NADH produced during glycolysis. A high rate of glycolysis during the de novo synthesis of cholesterol, for example, generates high levels of NADH. If the levels of NADH exceed the carrying capacity of the shuttle system, the reducing equivalents are transferred to pyruvate via the enzyme, lactate dehydrogenase. A high level of lactate is a signal that the shuttle system is rate limiting.

The ovary produces appreciable amounts of lactate, even during the early follicular phase [65]. As follicular size increases, lactate levels also increase [66], coinciding with the start of antrum formation and detectable estradiol-17β secretion [66,67]. After the LH surge, the levels of lactate increase an additional 2.5 fold [66,68]. In luteal tissue, a high percentage of the glucose taken up is metabolized only as far as pyruvate and lactate [69]. Iodoacetate, an inhibitor of glycolysis, abolishes the effect of LH on lactate accumulation and significantly reduces LH-stimulated progesterone synthesis [68,70].

The fact that the malate-aspartate shuttle is rate-limiting could be the reason for a mitochondrial location for HSD. However, pregnenolone is an end-product inhibitor of the P450_scc _reaction [71,72], and a mitochondrial location for HSD would remove the steroid from its site of inhibition. Progesterone does not inhibit the P450_scc _reaction [71]. Evidence that mitochondrial HSD is involved in the production of high levels of progesterone is provided by the observation that mitochondria from thecal tissue convert only 6.4 % of total ^14^C-cholesterol to ^14^C-progesterone (1.2 %) and ^14^C-pregnenolone (5.2 %); whereas, in contrast, mitochondria from luteal tissue convert 16.1 % of total ^14^C-cholesterol to ^14^C-progesterone (13.5 %) and ^14^C-pregnenolone (2.6 %) [73].

In human adrenals and gonads, HSD is derived from the same gene and has been classified as type II, relative to the placental enzyme, which is classified as type I [27,28,74,75]. In the adrenals and gonads of the rat [74-76] and mouse [77,78], the enzyme is also derived from the same gene, which in these rodents is classified as type I. The fact that ovaries and adrenal cortex contain the same HSD enzyme indicates that their response to trophic hormone stimulation would also be the same. One would expect, therefore, that ACTH stimulation would cause an increase in mitochondrial HSD activity in adrenal cortex tissue. ACTH stimulation of male and female rats does cause an increase in HSD mRNA and HSD activity [79]. However, it is not known if ACTH stimulation causes a preferential increase in mitochondrial HSD activity.

Aside from the role that Steroidogenic Acute Regulatory protein (StAR) plays in controlling cholesterol access to mitochondria, the rate-limiting step in steroidogenesis is considered to be P450_scc _[80]. However, the fact that P450_scc _and HSD are bound together as a complex [24] suggests that the rate-limiting step, or steps, entails the conversion of cholesterol to progesterone. There is a decided advantage in these two enzymes functioning as a unit. Instead of shuttling steroid intermediates from organelle to organelle, cholesterol can be converted to progesterone in what could be described as a single step. The levels of progesterone can be increased even further if the reactions of both enzymes are coupled together, which appears to be the case. The discovery of the energy-independent NADH/NADP^+ ^transhydrogenase initially led to the assumption that it existed to supply P450_scc _with NADPH [56]. However, the transhydrogenase is capable of producing less than one/half the NADPH needed to synthesize high levels of pregnenolone. If its function is to act as a principal supplier of NADPH, it is inadequate. However, if its main function is to ensure that HSD has an undiminished supply of NAD^+^, and is operating at V_max_, it is more than adequate. It is an ideal means of coupling HSD to P450_scc_. Unfortunately, the exchange of one molecule of NADH to produce one molecule of NADPH is insufficient for the overall conversion of progesterone to cholesterol. This is because the P450_scc _reaction utilizes 3 molecules of NADPH for the synthesis of one molecule of pregnenolone. The remainder of the NADPH for this reaction would have to be supplied by mitochondrial NADP^+^-linked isocitrate dehydrogenase [56].

**Figure 5:**
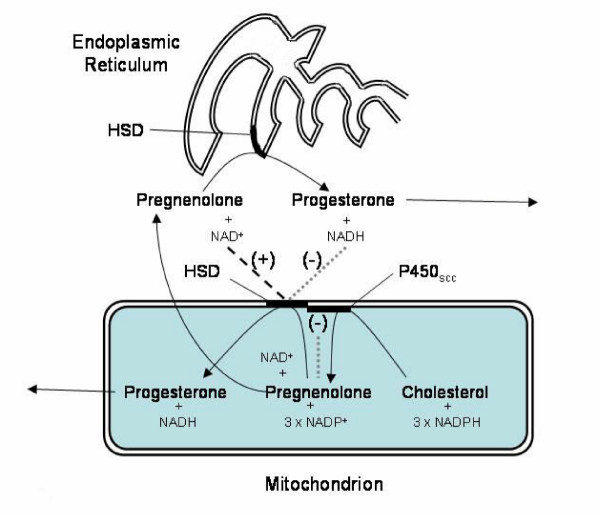
**The proposed regulation of progesterone synthesis by pregnenolone and by extramitochondrial NAD^+^/NADH**. In the overall conversion of cholesterol to progesterone, pregnenolone acts as an end product inhibitor in the P450_scc _reaction, [**······ (-)**]; and, in the mitochondrial HSD reaction, extramitochondrial NAD^+ ^operates as an allosteric activator [**------(+)**] and extramitochondrial NADH operates as an allosteric inhibitor [**······ (-)**].

The P450_scc_/HSD enzyme complex is well regulated. In addition to the end-product inhibition exerted by pregnenolone on the P450_scc _reaction [71,72], the HSD reaction is affected by the redox state of cytoplasmic pyridine nucleotides [81]. For example, extramitochondrial NAD^+ ^increases mitochondrial HSD activity by up to 40 %; whereas, extramitochondrial NADH inhibits HSD activity by as much as 70 %. Figure 5 is a representative diagram of the proposed regulation of the conversion of cholesterol to progesterone. As indicated, pregnenolone acts as an end product inhibitor of the P450_scc _reaction, [**······ (-)**]; and, in the mitochondrial HSD reaction, extramitochondrial NAD^+ ^operates as an allosteric activator [**------(+)**] and extramitochondrial NADH operates as an allosteric inhibitor [**······ (-)**]. With this level of control it is difficult to imagine how pregnenolone could ever leave the luteal cells without being converted to progesterone.

Finally; it was noted in the results section that total ovarian protein was inversely correlated with the peaks of LH. A decrease in total ovarian protein during diestrus could be due to ovulation. However, this does not explain the lower protein levels following LH stimulation that occurs during pregnancy and after parturition. A rapid and ongoing synthesis of the two enzymes, P450_scc _and HSD is critical to the production of high levels of progesterone. This necessitates a ready supply of amino acids. It is speculative of course, but the action of LH could include the initiation of proteolysis of protein stores from luteal tissue, which could explain the lower protein levels.

## Conclusion

The ovary has two levels of steroid synthesis. One level occurs during the follicular phase, and a higher level of synthesis occurs throughout the luteal phase. We believe the higher level of synthesis is due to, and the reason for, mitochondrial HSD. To synthesize estradiol-17β during the follicular phase, steroid precursors are shuttled from cell type to cell type and from organelle to organelle. The synthesis of progesterone during the luteal phase involves one cell type and two enzymes. With HSD in the mitochondria, rather than in the microsomes, the shuttle of steroid precursors is unnecessary. It also allows for HSD and P450_scc _to function together as a unit, a decided advantage for producing high levels of progesterone. This is especially true if the two enzymes are coupled together by the NADH/NADP^+ ^transhydrogenase. A mitochondrial location for HSD also solves the problem inherent with the rate-limiting malate-aspartate shuttle, and it removes the end-product inhibition of pregeneolone by converting it to progesterone. Finally, the fact that HSD and P450_scc _have a strong binding affinity for each other, and are synthesized simultaneously, tentatively suggests a means by which LH stimulation results in an increase in mitochondrial HSD activity.
